# Comparison of PPAR Ligands as Modulators of Resolution of Inflammation, via Their Influence on Cytokines and Oxylipins Release in Astrocytes

**DOI:** 10.3390/ijms21249577

**Published:** 2020-12-16

**Authors:** Dmitry V. Chistyakov, Alina A. Astakhova, Sergei V. Goriainov, Marina G. Sergeeva

**Affiliations:** 1 Belozersky Institute of Physico-Chemical Biology, Lomonosov Moscow State University, 119992 Moscow, Russia; alina_astakhova@belozersky.msu.ru (A.A.A.); mg.sergeeva@gmail.com (M.G.S.); 2 SREC PFUR, Peoples’ Friendship University of Russia (RUDN University), 117198 Moscow, Russia; goryainovs@list.ru

**Keywords:** peroxisome proliferator-activated receptors (PPAR), rosiglitazone, GW9662, fenofibrate, GW6471, GW501516, GSK0660, toll-like receptors (TLRs)

## Abstract

Neuroinflammation is a key process of many neurodegenerative diseases and other brain disturbances, and astrocytes play an essential role in neuroinflammation. Therefore, the regulation of astrocyte responses for inflammatory stimuli, using small molecules, is a potential therapeutic strategy. We investigated the potency of peroxisome proliferator-activated receptor (PPAR) ligands to modulate the stimulating effect of lipopolysaccharide (LPS) in the primary rat astrocytes on (1) polyunsaturated fatty acid (PUFAs) derivative (oxylipins) synthesis; (2) cytokines TNFα and interleukin-10 (IL-10) release; (3) p38, JNK, ERK mitogen-activated protein kinase (MAPKs) phosphorylation. Astrocytes were exposed to LPS alone or in combination with the PPAR ligands: PPARα (fenofibrate, GW6471); PPARβ (GW501516, GSK0660); PPARγ (rosiglitazone, GW9662). We detected 28 oxylipins with mass spectrometry (UPLC-MS/MS), classified according to their metabolic pathways: cyclooxygenase (COX), cytochrome P450 monooxygenases (CYP), lipoxygenase (LOX) and PUFAs: arachidonic (AA), docosahexaenoic (DHA), eicosapentaenoic (EPA). All tested PPAR ligands decrease COX-derived oxylipins; both PPARβ ligands possessed the strongest effect. The PPARβ agonist, GW501516 is a strong inducer of pro-resolution substances, derivatives of DHA: 4-HDoHE, 11-HDoHE, 17-HDoHE. All tested PPAR ligands decreased the release of the proinflammatory cytokine, TNFα. The PPARβ agonist GW501516 and the PPARγ agonist, rosiglitazone induced the IL-10 release of the anti-inflammatory cytokine, IL-10; the cytokine index, (IL-10/TNFα) was more for GW501516. The PPARβ ligands, GW501516 and GSK0660, are also the strongest inhibitors of LPS-induced phosphorylation of p38, JNK, ERK MAPKs. Overall, our data revealed that the PPARβ ligands are a potential pro-resolution and anti-inflammatory drug for targeting glia-mediated neuroinflammation.

## 1. Introduction

Astrocytes are glial cells that are important players in the development of neuroinflammation [1,2,3,4]. Neuroinflammation accompanies all known neurological pathologies, including neurodegenerative diseases, post-ischemic neurodegeneration, as well as traumatic, metabolic, toxic and neoplastic disturbances [2,5,6]. Therefore, significant effort is being applied to understand the mechanisms of the inflammatory response on astrocytes and to select substances that can control this process.

During the last decade, a significant breakthrough was made in relation to understanding the molecular mechanisms of inflammation, and it was shown that unbalanced positive and negative feedback loops of innate immune regulatory pathways, can result in chronic inflammation [7,8,9]. Special attention was given to the resolution of inflammation processes, since manipulation of these processes may be the key to treating pathologies, associated with chronic inflammation [8,9,10]. Besides cytokines, such as interleukin-10 (IL-10) [11], the processes of resolution are mainly associated with lipid mediators, named oxylipins, that are derivatives of ω-3 and ω-6 polyunsaturated fatty acid (PUFAs), such as arachidonic (AA), docosahexaenoic (DHA) and eicosapentaenoic (EPA) acids [12,13]. Oxylipins are synthesized from PUFAs via multiple oxidative reactions, catalyzed by specific enzymes (cyclooxygenases (COXs), lipoxygenases (LOXs) or cytochrome P450 monooxygenases (CYP)), or proceeding in an enzyme-independent manner in the presence of reactive oxygen species (ROS) [14]. It is generally considered that pathology in inflammatory processes is closely associated with an imbalance between ω-3 (DHA, EPA) and ω-6 (AA) PUFAs and their metabolism, which leads to the hyperproduction of pro-inflammatory lipid mediators (ω-6 derivative oxylipins, such as prostaglandins) and the underproduction of pro-resolving (ω-3 derivative oxylipins) molecules [12]. Although the functional significance of oxylipins was known long ago, research in this field has not been sufficiently intensive, due to the absence of high-throughput methods for the detection of these molecules. The development of mass spectrometry has greatly increased knowledge relating to the lipid mediators of inflammation and resolution [15,16,17,18]. These methods make it possible to raise the question of a targeted search for substances that can shift the oxylipin profiles towards resolution.

The ligands of peroxisome proliferator-activated receptor (PPARs) occupy a special place among substances that can regulate the balance between various oxylipins, and thus, shift inflammatory processes towards resolution. There are several reasons for this. The PPARs are ligand-activated transcription factors of the nuclear receptor superfamily. Three PPAR subtypes exist, encoded by separate genes (α, NR1C1; β/δ, NR1C2 and γ, NR1C3). Upon ligand binding, PPARs activate target gene transcription and regulate various important physiological processes, such as lipid metabolism, inflammation and wound healing [6,19]. The subtypes possess a high degree of structural homology and share the same DNA response element (PPRE, the PPAR response element) [20]. Part of the PUFAs metabolizing genes include PPRE in their promoters [20]. Moreover, PUFAs and certain oxylipins are endogenous agonists of PPARs [21]. Therefore, multiple positive and negative feedback regulatory pathways may be involved in PPAR activation, by exogenously added ligands [21]. Understanding the mechanisms of these substances’ action is complicated by the fact that depending on the context, PPARs can interfere with other transcription factors, such as NF-kB, and they also have nongenomic actions [20,22]. Importantly, PUFAs and certain oxylipins are natural endogenous agonists of PPARs [21], while some synthetic PPAR agonists are well-known drugs [20]. PPARα targets the fibrate class of hypolipidemic drugs and PPARγ for the thiazolidinediones class of insulin-sensitizing drugs [20]. The application of synthetic PPAR ligands for the regulation of inflammatory processes implies knowledge about the interplay mechanisms between endogenous and synthetic ligands, which is still meager.

Recent studies assessing the neuroprotective efficacy of synthetic PPARα, -β, or -γ agonists indicate that they may ameliorate clinical symptoms and reduce the severity of a variety of acute and chronic brain pathologies, associated with inflammatory responses [23,24,25,26,27,28]. As a key regulator of the innate immune system, the interaction between PPARs and TLRs has been suggested as a potential therapeutic target in disease treatment [29]. In addition, it was suggested that PPARγ agonists potentiated an anti-inflammatory M2 glial phenotype [30]. Although, all three PPARs have been shown to interact [3], the effects of synthetic PPAR ligands usually are evaluated separately (see, for example, references in [6]). Taken together, these findings provided the impact for our comparison of the efficacy of all three types of PPAR ligands as modulators of oxylipin profiles and cytokines production in the course of TLR-mediated astrocytes responses.

In our work, we have selected the most commonly used ligands for each PPAR subtype, which have the most promising therapeutic potential [3,6,21,25,26,27,28,29,30,31]. These ligands include the PPARα agonist, fenofibrate and the antagonist GW6471; the PPARβ agonist, GW501516 and the PPARβ antagonist and the inverse agonist, GSK0660; the PPARγ agonist, rosiglitazone and the antagonist, GW9662. We used a cellular model of rat primary astrocytes, stimulated by LPS. Previously it was shown that LPS activated TLR4 in astrocytes [31]. We included agonist-antagonist pairs specific for each PPAR subtype in the subtype comparison. This was done because there is ample evidence of antagonists’ independent effects at the cellular level [3,6,25]. These effects may be associated with the peculiarities of the interaction of endogenous (PUFAs, oxylipins) and exogenous ligands of PPAR during the development of cellular response to a stimulus. We compared the PPAR ligands by their modulation of LPS which induced (1) oxylipin synthesis; (2) cytokine release (TNFα and interleukin 10) and (3) phosphorylation of p38, JNK, ERK mitogen-activated protein kinases (MAPKs). The data reveal that the PPARβ ligands, GW501516 and GSK0660 are the substances with more anti-inflammatory and pro-resolution features.

## 2. Results

### 2.1. PPARs Ligands’ Effects on the Synthesis of Oxylipins by Naive and LPS-Stimulated Astrocytes

Oxylipins release accompanies LPS-mediated astrocytes responses [32,33,34]. Although measuring oxylipin profiles was suggested to be important in estimating astrocyte features in inflammation [35], there were no data concerning PPAR ligands. Therefore, we compared the ability of the PPAR ligands to modulate LPS-mediated astrocyte responses and influence oxylipin profiles in naive astrocytes. We used LPS treatment for 4 h. The ligands were added 30 min before LPS (100 ng/mL) stimulations and oxylipin profiles were measured in a culture medium by UPLC-MS/MS lipidomic analysis (see details in the Section 4). We examined the effect of the tested agonists in pairs indicated by their main targets-agonists and antagonists of PPAR subtypes. We also evaluated the change in their effects when they were added together.

#### 2.1.1. Comparison of PPARα Ligands: Agonist Fenofibrate (Fen), Antagonist GW6471 (GW6)

**Figure 1:**
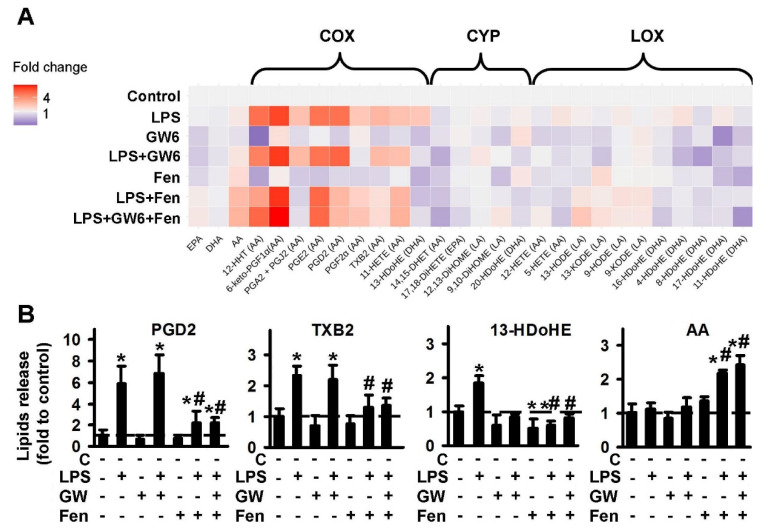
Effect of PPARα agonist fenofibrate and antagonist GW6471 on the oxylipins release in the LPS-stimulated astrocytes. Primary rat astrocytes were pretreated for 30 min with GW6471 (GW6, 5 μM) or Fenofibrate (Fen, 50 μM) or in combination, and then stimulated with LPS (100 ng/mL) for 4 h. Concentrations of oxylipins in supernatants were measured using UPLC-MS/MS. (**A**) The heat map shows relative amounts of each lipid mediator compared to the control. The vertical axis indicates the stimuli, while the horizontal axis indicates the relative amount (log2) of each lipid mediator. Metabolites were divided into: Lipoxygenase (LOX), cyclooxygenase (COX), and cytochrome (CYP) pathways involved in their synthesis. (**B**) The bars show relative amounts of COX-derived lipid mediators. Values represent the mean ± SEM from three independent experiments. * *p* < 0.05, compared with the unstimulated cells, # *p* < 0.05, compared with the LPS-stimulated cells.

We identified 28 compounds, which were classified according to their metabolic pathways: cyclooxygenase (COX), cytochrome P450 monooxygenases (CYP) and lipoxygenase (LOX); PUFAs were also determined: AA, DHA, EPA (Figure 1A). The data are represented as a heat map, with the vertical axis indicating the stimuli and the horizontal axis indicating the relative amount of each lipid mediator (quantitative data presented in Figure S1).

The PPARα agonist, fenofibrate decreases the LPS-stimulated synthesis of the COX-metabolized substances: 12-HHT, PGD2, PGA2 + PGJ2, TXB2, 13-HDoHE. Fenofibrate also increases the release of extracellular AA. PPARα antagonist GW6471 possesses its own activity via inhibition of the CYP-metabolized substances, 14,15-DHET, 20-HDoHE. GW6471 does not modulate COX-metabolized derivatives or AA release. The representative data for treatments are presented in Figure 1B. It is notable that there is no action as classical agonist-antagonist pairs, in treatments where both PPARα ligands were added simultaneously (Figure 1B, Figure S1). Although it is possible to suppose fenofibrate is an anti-inflammatory modulator due to its activity, as an inhibitor of LPS-mediated prostaglandin synthesis, the effect does not seem to have been realized via PPARα, as the antagonist, GW6471 did not reverse it.

#### 2.1.2. Comparison of PPARβ Ligands: Agonist GW501516 (GW5), Antagonist and Inverse agonist GSK0660 (GSK)

**Figure 2:**
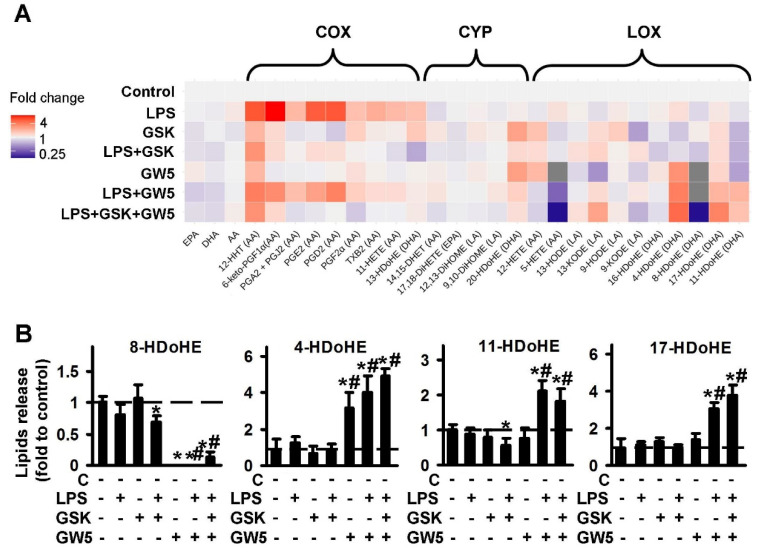
Effect of PPARβ agonist GW501516 and antagonist GSK0660 on the oxylipins release in the LPS-stimulated astrocytes. Primary rat astrocytes were pretreated for 30 min with GSK0660 (GSK, 5 μM) or GW501516 (GW5, 25 μM) or in combination, and then stimulated with LPS (100 ng/mL) for 4 h. Concentrations of oxylipins in supernatants were measured using UPLC-MS/MS. (**A**) The heat map shows relative amounts of each lipid mediator compared to the control. The vertical axis indicates the stimuli, while the horizontal axis indicates the relative amount (log2) of each lipid mediator. Metabolites were divided into: Lipoxygenase (LOX), cyclooxygenase (COX), and cytochrome (CYP) pathways involved in their synthesis. (**B**) The bars show relative amounts of COX-derived lipid mediators. Values represent the mean ± SEM from three independent experiments. * *p* < 0.05, compared with the unstimulated cells, # *p* < 0.05, compared with the LPS-stimulated cells.

During an investigation into the involvement of PPARβ in LPS-mediated oxylipin synthesis in astrocytes, we used the agonist, GW501516 and substance, GSK0660, which is commonly used as an antagonist of PPARβ, but also considered as an inverse agonist of PPARβ [36]. The data are represented as a heat map (Figure 2A). The quantitative data are presented in Figure S2. Both PPARβ ligands inhibit LPS-stimulated oxylipins’ synthesis via the COX pathway, and GSK0660 is a stronger inhibitor than GW501516, in relation to the concentrations used (Figure 2A). Inhibited: 12-HHT; 6-keto-PGF1a, PGA2 + PGJ2, PGE2, PGD2, PGF2a, TxB2, 11-HETE, 13-HDoHE. It is worthy of note that adding GSK0660 increases the synthesis of 13-HDoHE, 12-HHT and PGF2a, that may reveal the synthesis of these substances via other metabolic pathways [37,38,39]. Such modulation also allows us to consider the PPARβ ligand, GSK0660 as an inverse agonist, not antagonist, in our tested model. Besides the COX pathway, the PPARβ agonist, GW501516, decreases LPS-mediated oxylipins, attributed to the LOX-metabolized pathway: 5-HETE, 8-HDoHE, and significantly increases the synthesis of 4-HDoHE, 11-HDoHE, 17-HDoHE (Figure 2B). The last three substances are considered to be important as a “substance of resolution” of inflammation [40,41], while 5-HETE and 8-HDoHE have proinflammatory features [42,43]. It is noted that 4-HDoHE, 8-HDoHE, 11-HDoHE, 13-HDoHE and 17-HDoHE are derivatives of DHA, while both tested PPARβ ligands do not influence the concentration of extracellular PUFAs (DHA, AA, EPA) (see details in Figure S2). Overall, the data show that both PPARβ ligands tested, have the potential to decrease LPS-mediated prostaglandin synthesis; among them, agonist GW501516 is a strong inducer of pro-resolution substances.

#### 2.1.3. Comparison of PPARγ Ligands: Agonist Rosiglitazone (RG) and Antagonist GW9662 (GW9)

**Figure 3:**
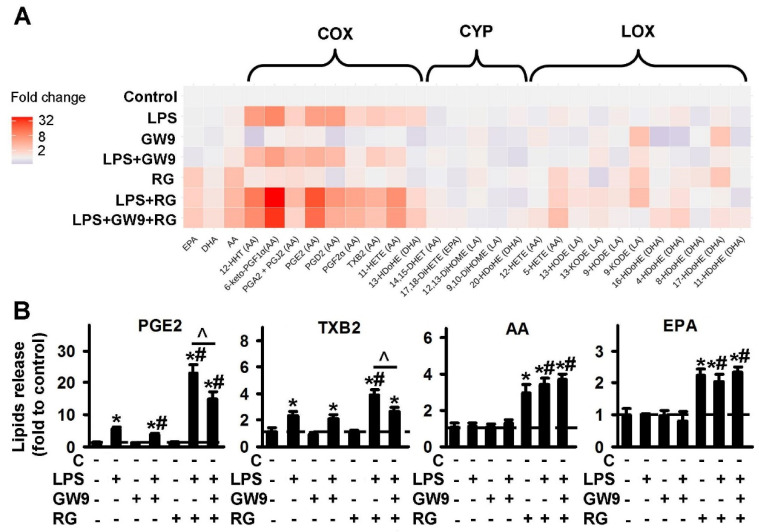
Effect of PPARγ agonist rosiglitazone and antagonist GW9662 on the oxylipins release in the LPS-stimulated astrocytes. Primary rat astrocytes were pretreated for 30 min with GW9662 (GW9, 5 μM) or rosiglitazone (RG, 20 μM) or in combination, and then stimulated with LPS (100 ng/mL) for 4 h. Concentrations of oxylipins in supernatants were measured using UPLC-MS/MS. (**A**) The heat map shows relative amounts of each lipid mediator compared to the control. The vertical axis indicates the stimuli, while the horizontal axis indicates the relative amount (log2) of each lipid mediator. Metabolites were divided into: Lipoxygenase (LOX), cyclooxygenase (COX), and cytochrome (CYP) pathways involved in their synthesis. (**B**) The bars show relative amounts of COX-derived lipid mediators. Values represent the mean ± SEM from three independent experiments. * *p* < 0.05, compared with the unstimulated cells, # *p* < 0.05, compared with the LPS-stimulated cells, ^ *p* < 0.05, compared with the indicated treatment.

For an investigation into the involvement of PPARγ in oxylipin synthesis in astrocytes, we used the agonist, rosiglitazone and the classical antagonist, GW9662 [44]. The data are represented as a heat map (Figure 3A). The quantitative data are presented in Figure S3. Both PPARγ ligands demonstrate the influence on oxylipin synthesis in naive astrocytes. Rosiglitazone increases the LPS-stimulated synthesis of oxylipins from AA via the COX pathway. The effect was antagonized by GW9662 (Figure 3B, Figure S3). Rosiglitazone also increases concentrations of the extracellular PUFAs, AA and EPA, but not DHA, both in naive and LPS-stimulated cells (Figure 3B). This effect is not reversed by GW9662 (Figure 3B). The simultaneous addition of both PPARγ ligands increases the LPS-mediated synthesis of 5-HETE and 4-HDoHE (Figure S3).

### 2.2. PPARs Ligands as Modulators of COX-2 Expression

**Figure 4:**
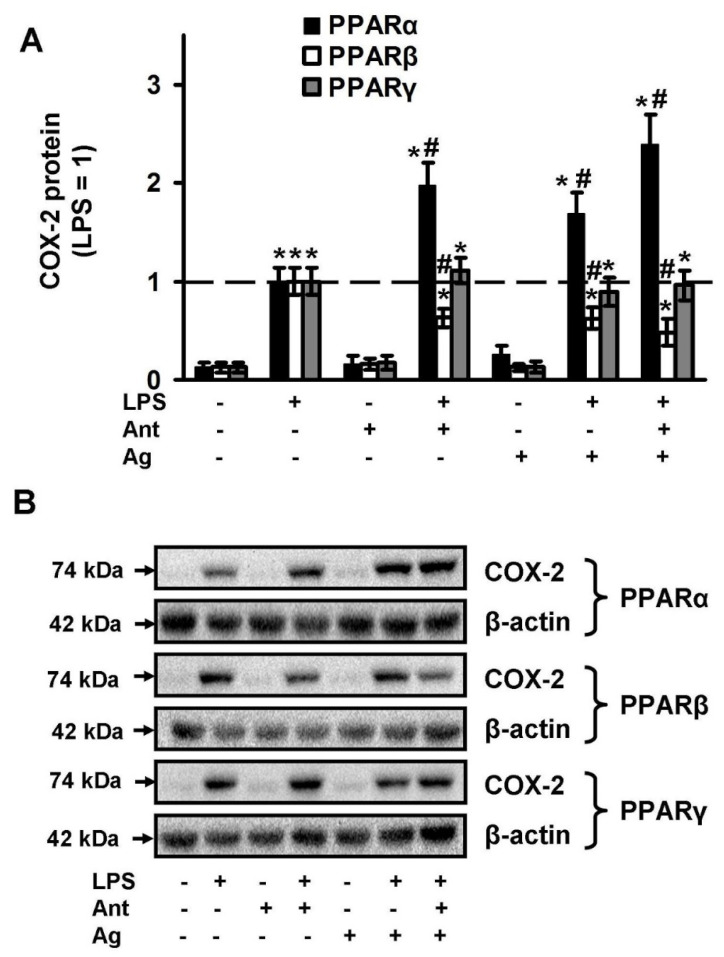
Effects of PPARs ligands on the expression of COX-2 upon stimulation with LPS. Astrocytes were pretreated with PPARα agonist Fenofibrate (50 μM), PPARα antagonist GW 6471 (5 μM), PPARβ agonist GW501516 (25 μM), PPARβ antagonist: GSK0660 (5 μM), PPARγ agonist Rosiglitazone (20 μM), PPARγ antagonist GW9662 (5 μM) for 30 min and then stimulated with LPS (100 ng/mL) for 4 h. COX-2 protein levels were evaluated by western blotting and normalized to the loading control β-actin. (**A**) Representative Western blots demonstrating COX-2 protein levels. (**B**) Results are expressed as fold-changes relative to LPS-treated astrocytes. The example is representative for three independent experiments. Values represent mean ± SEM from three independent experiments. * *p* < 0.05, compared with the unstimulated cells, # *p* < 0.05 compared with the LPS-stimulated cells.

The obtained oxylipin profiles reveal that all tested PPAR ligands modulate the LPS-stimulated synthesis of PUFA derivatives via the COX-dependent pathway. We have previously shown that COX-2 is the key enzyme, responsible for the LPS-induced synthesis of appropriate substances in astrocytes [45,46]. Therefore, we analyzed the modulation of LPS-induced COX-2 expression by tested PPAR ligands (Figure 4). We found that the substances do not influence the COX-2 protein level in naive cells, but PPARα and PPARγ ligands modulate LPS-stimulated COX-2 protein expression (Figure 4). Both the PPARα agonist, fenofibrate and the antagonist, GW6471 increase the protein level two-fold. In contrast, both PPARβ ligands (agonist GW501516 and antagonist GSK0660) decrease the LPS-mediated COX-2 expression (Figure 4), which may, in part, explain their inhibitory effect on the COX-dependent prostaglandin synthesis.

### 2.3. PPAR Ligands as Modulators of p38, JNK, ERK1\2 Mitogen Activated Protein Kinases (MAPKs)

**Figure 5:**
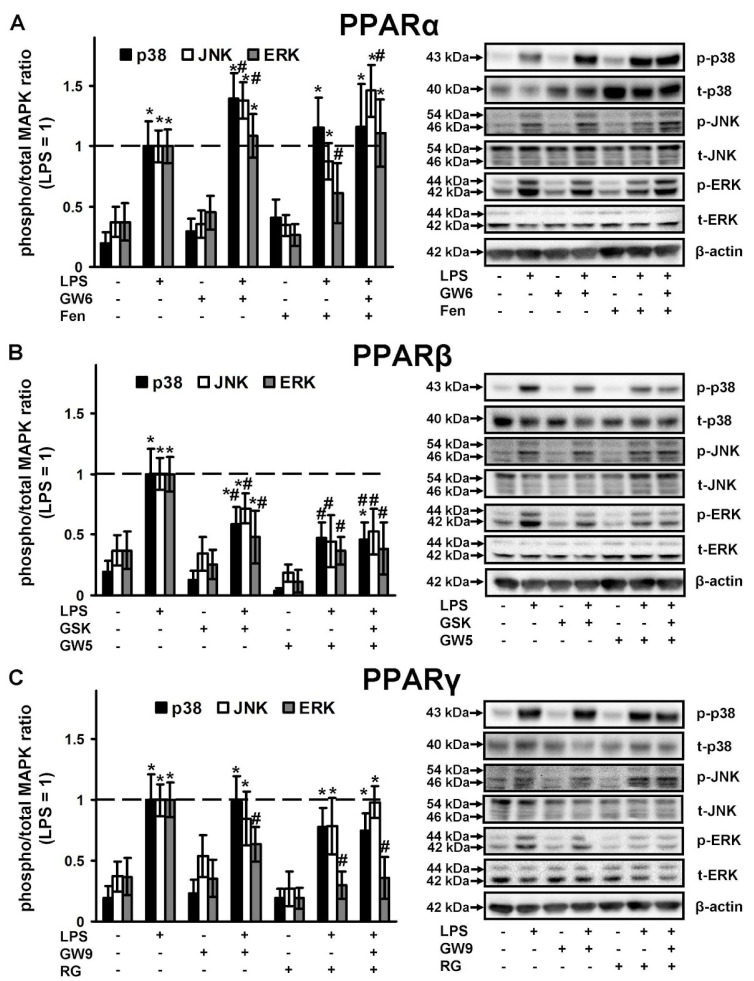
Comparison of p38, JNK and ERK1/2 MAPK activity in the LPS-stimulated astrocytes, treated with PPARs ligands. Astrocytes were pretreated with PPARα agonist Fenofibrate (50 μM), PPARα antagonist GW 6471 (5 μM), PPARβ agonist GW501516 (25 μM), PPARβ antagonist: GSK0660 (5 μM), PPARγ agonist Rosiglitazone (20 μM), PPARγ antagonist GW9662 (5 μM) for 30 min and then stimulated with LPS (100 ng/mL) for 4 h. p38, p-p38, pJNK, JNK, pERK1/2 and ERK1/2 protein levels were evaluated by western blotting and normalized to the loading control β-actin. Representative results are expressed as fold-changes relative to LPS-treated astrocytes. Western blots for (**A**) PPARα ligands, (**B**) PPARβ ligands, (**C**) PPARγ ligands demonstrating MAPKs protein levels. Values represent mean ± SEM from three independent experiments. * *p* < 0.05, compared with the unstimulated cells, # *p* < 0.05 compared with the LPS-stimulated cells.

The three major groups of regulated MAPK cascades that lead to altered gene expression and modulate signaling at transcriptional and post-transcriptional levels, are well-documented: p38, JNK, ERK1/2 MAPKs [47]. MAPKs are components of TLR-mediated signaling and their roles in astrocytes are being thoroughly investigated [31,48,49]. MAPK inhibitors are viewed as potential anti-inflammatory agents in brain disturbances [47]. Therefore, we compared PPAR ligands as the inhibitors of the activity of these MAPKs in naive and LPS-stimulated astrocytes. All three MAPKs tested were activated in the presence of LPS (Figure 5). The PPARα agonist, fenofibrate, when added to naive cells decreases ERK activity and slightly increases p38 and ERK activity in LPS-stimulated cells (Figure 5A). The PPARα antagonist, GW6471 does not influence naive cells but increases p38, JNK and ERK activity in the presence of LPS (Figure 5A). Both PPARβ ligands do not influence MAPKs activity in naive cells, but significantly decrease the LPS-mediated activity of all measured MAPKs (p38, JNK, ERK) (Figure 5B). Both PPARγ ligands only have the effect of inhibiting LPS-mediated ERK activity, but not p38 or JNK (Figure 5C). Overall, our data reveal that PPARβ ligands are more effective anti-inflammatory substances by comparison with other types of PPAR ligands.

### 2.4. Effects of PPAR Ligands on Cytokine Markers of Inflammation (TNFα and IL-10)

**Figure 6:**
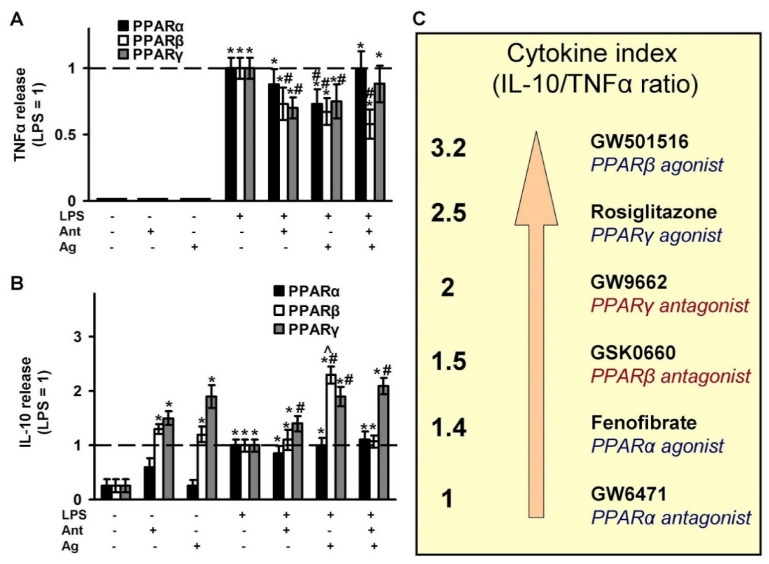
Effect of PPARs ligands on the inflammatory response. The primary rat astrocyte cultures were pretreated with PPARα agonist Fenofibrate (50 μM), PPARα antagonist GW 6471 (5 μM), PPARβ agonist GW501516 (25 μM), PPARβ antagonist: GSK0660 (5 μM), PPARγ agonist Rosiglitazone (20 μM), PPARγ antagonist GW9662 (5 μM) for 30 min and then stimulated with LPS (100 ng/mL) for 4 h. The TNFα (**A**) and IL-10 (**B**) protein release measured by ELISA in supernatant samples. The results are expressed as fold-changes, relative to LPS-stimulated cells. The values represent a mean ± SEM from three independent experiments. * *p* < 0.05, compared with the non-stimulated cells, # *p* < 0.05, compared with the LPS-stimulated cells, ^ *p* < 0.05 compared with the agonist-treated cells. (**C**) Cytokine index as a ratio IL-10 and TNFα allows to line up a row for the tested substances relative to their anti-inflammatory and resolution features.

To assess the effectiveness of the tested ligands in terms of their effect on cytokine synthesis, we chose TNFα as an inflammatory marker on astrocytes [49,50] and IL-10 as an anti-inflammatory and pro-resolution marker [11,51]. The concentrations of cytokines released under the LPS stimulation were determined in parallel with oxylipins in the same experimental cellular cultures. TNFα is an inducible cytokine for astrocytes, thus, without the addition of LPS, we did not observe the effect of the tested substances on the induction of the cytokine (Figure 6A). LPS stimulates the release of almost 400 pg/mL TNFα in 4 h, and the concentration was taken as 1 (Figure 6A). The LPS-stimulated release of TNFα was inhibited by the agonists of all three receptors (PPARα, -β, -γ), and also by the PPARβ and PPARγ antagonists. The co-additions of test substances as agonist and antagonist pairs have been shown only to abolish the PPARα agonist effect. IL-10 is considered an anti-inflammatory cytokine and is even referred to as a cytokine of resolution [11]. The level of IL-10 in naive astrocytes is near 25 pg/mL. LPS induced IL-10 synthesis three-fold (taken as 1 in Figure 6B). The tested pairs of PPARβ and PPARγ increased IL-10 release even more than LPS in the naive cells. The data suggest that these substances can be classified as protective substances that may increase the level of the anti-inflammatory cytokine, which, in turn, can protect cells from contact with an inflammatory agent. The effects persisted with the addition of LPS (Figure 6B). Moreover, the stimulation by LPS even induced the effect of the PPARβ agonist (Figure 6B). The level of IL-10 following co-treatment of the stimuli (LPS) and the PPARβ agonist was abolished in the presence of the PPARβ antagonist (Figure 6B) which suggests the involvement of PPARβ in this effect. Test substances from a PPARα pair did not affect the IL-10 level, both in naive and LPS-stimulated cells (Figure 6B). A substance can be considered the better “resolution stimuli”, the more it inhibits the LPS-stimulated release of the pro-inflammatory cytokine (TNFα) and activates the release of the anti-inflammatory cytokine (IL-10). Therefore, we calculated the cytokines index (IL-10/TNFα) (Figure 6C). The best substance is GW501516, a PPARβ agonist. The test substances are arranged in the following order (Figure 6C): GW501516 > Rosiglitazone > GW9662 > GSK0660 > Fenofibrate > GW6471. It is to be noted that although there is no strict relationship between the PPAR subtype pairs, agonist-antagonist, the PPARα subtype pair substances have a weaker anti-inflammatory effect regarding the synthesis of cytokines in our cell model. At the same time, PPARβ and PPARγ can be viewed as substances that facilitate the inflammatory process via anti-inflammatory and resolution processes.

## 3. Discussion

Our data confirm the previously suggested hypothesis that the use of selective PPAR subtype ligands may represent an opportunity to develop new therapeutic strategies for traumatic brain injuries and neurodegenerative diseases [6]. Today, it is accepted that the inflammation processes, mediated by astrocytes, affect animal and human behavior, including pain, appetite, sleep, mood and disease symptoms [4]. Astrocytes contain all components of the TLR signaling pathways, which take an active part in neuroinflammation [52,53,54]. Three PPAR subtypes have been reported in astrocytes [55] and their involvement in LPS-stimulated inflammatory response investigated in various cellular models [48,56,57]. We concluded that all three PPAR subtype ligands are powerful anti-inflammatory substances at the level of the astrocytes’ cellular model. They modulate LPS-stimulated cells’ responses through (1) influence on oxylipins’ synthesis, (2) inhibition of MAPKs activity and (3) the effect on the release of cytokines.

The involvement of PUFAs and their metabolites in the regulation of brain function and disease have been discussed over a long period (reviewed in [58]). It is generally accepted that the prostaglandins, AA-derivatives via COX pathway, are associated with neuropsychiatric and neurological disorders [58,59]. Prostaglandins promote cytokine production, and the cytokines produced are harmful to neurons [60]. From that point of view, all tested substances, besides rosiglitazone, can be identified as anti-inflammatory, as they inhibit LPS-mediated oxylipin synthesis via COX pathways. By decreasing the order of activity, substances can be arranged as follows: GSK0660 (antagonist PPARβ) > GW501516 (agonist PPARβ) > GW9662 (antagonist PPARγ) >> fenofibrate (agonist PPARα) > GW6471 (antagonist PPARα). Moreover, the PPARβ agonist, GW501516, increased the synthesis of 4-HDoHE, 11-HDoHE and 17-HDoHE, the derivatives of DHA, indicating that they are substances of resolution of inflammation [40,41]. The activation of prostaglandin by rosiglitazone has been identified [56]. However, there is still doubt as to whether we can attribute the effect to the side feature of rosiglitazone. These doubts are due to the fact that the role of prostaglandins as only pro-inflammatory substances has not been determined. For example, it was shown that PGE2 protected neurons from LPS-induced apoptosis, via modulation of ROS production [61]. We have shown that using various ligands of PPARs, and it is possible to manipulate oxylipins’ metabolism. Although it is difficult to predict the effect of such manipulations at present, it is important to note that many oxylipins are released in low concentrations, their effects can be summarized [35]. It follows that neuroprotective properties should be tested not for individual oxylipins but their mixtures. Nevertheless, according to the existing views on the role of prostaglandins, it is the ligand, PPARβ GW501516 that can be considered a more successful modulator of inflammatory processes on astrocytes, as part of LPS-induced oxylipin synthesis.

In response to pro-inflammatory stimuli, cytokine synthesis is an important stage in developing the cellular inflammatory response. Astrocytes, as well as microglia, respond to inflammatory stimuli (LPS, IL-1β, TNF) by the production of pro- and anti-inflammatory cytokines both in vitro and in vivo [35,53,62,63]. Our data are consistent with the former, in which the PPARα agonist, fenofibrate [57] and the PPARγ agonist, rosiglitazone [64] inhibited LPS-induced TNFα release in astrocytes. The PPARβ agonist, GW501516 decreased LPS-induced mRNA TNFα release in mixed brain cell cultures [36]. There are no data concerning the modulation of IL-10 release in astrocytes by PPAR ligands, except [65] and [53], where the effects of rosiglitazone both on IL-10 and TNFα release of LPS-stimulated astrocytes are consistent with our present data. The emergence of ideas about the resolution of inflammation draws our attention to solving the problem of inhibiting the synthesis of proinflammatory cytokines (TNFα, IL-6, etc.) and stimulating the synthesis of resolution cytokines (IL-10, IL-4, etc.). Therefore, we suggested using the IL-10/TNFα index for evaluating the tested substances. According to this index, the test ligands are arranged in the following order: GW501516 (PPARβ) > Rosiglitazone (PPARγ) > GW9662 (PPARγ) > GSK0660 (PPARβ) > Fenofibrate (PPARα) > GW6471 (PPARα). A good index for rosiglitazone indicates a positive role in the regulation of inflammation. Note that we obtained clear “agonist-antagonist” effects on the release of TNFα for PPARα, on the release of IL-10 for PPARβ. This means that in the LPS-treated cellular model, we see the summarized result of several different mechanisms modulated by PPAR ligands. Somewhere, antagonists act as antagonists or inverse agonists. Somewhere, perhaps, the substances do not act as ligands of PPAR (as has already been shown, for example, for rosiglitazone [66]). Further research is needed for the estimation of molecular mechanisms of these processes.

Although we did not find data concerning the comparison of all PPAR subtype ligands, nevertheless, there are certain data concerning the involvement of PPAR ligands in the modulation of innate immune responses in other cell types. It is not easy to compare TLR-mediated responses in astrocytes, macrophages and monocytes due to the difference in cells origin that realized on specificity in the regulation of TLR-mediated responses in astrocytes [48,49,53], but certain data can be considered. Firstly, the effects of the so-called PPAR antagonists themselves. Indeed, in relation to the cells of macrophage origin (RAW 264.7), all three PPAR subtype antagonists (GW5417; GSK0660; GW9662) increased TNFα synthesis by 12 h in the LPS-stimulated cells [67]. We identified that GSK0660 and GW9662 inhibited TNFα synthesis by 6 h on LPS-stimulated astrocytes. We did not find real agonist-antagonist pair effects for the three PPAR subtypes in the tested cellular model system. The antagonists’ opportunity to realize their own effects may be explained by the interference of exogenously added substances with the LPS-activated release of the endogenous substances, PUFAs and their derivatives [21,68]. This should be taken into account when considering the mechanisms of action of these substances. It was also shown that the long-term application (three days) of the PPARγ antagonist, GW9662, inhibited LPS-stimulated TNFα or IL-10 release at 48 h of LPS stimulation in cultured human monocytes [69]. Similar data were obtained with the long-term application of GW9662 on adipocytes [70]. Therefore, not only cell types, but also the time of application should be taken into consideration in future investigations of PPAR ligand mechanisms.

TLR-mediated signaling pathways include activation of MAPK cascades [47,71,72]. There is an amount of data concerning PPAR ligands and p38, JNK, ERK MAPK activity (see, for example, review [73]). The connections depend on the cell type, stimuli and time of application. In addition, there is a set of data concerning the non-genomic mechanisms of PPAR ligand-induced MAPK phosphorylation that might be involved in the consideration of PPAR ligand mechanisms [22]. Nevertheless, in general, MAPK inhibitors are viewed as potential anti-inflammatory agents in brain disturbances [47]. The increased activity of MAPKs in activated astrocytes, and their regulatory role in the synthesis of inflammatory cytokines mediators, make them potential targets for novel therapeutics [47]. Within this framework and, together with our data, the PPARβ ligands are once again better than the tested ligands of other PPAR types.

## 4. Materials and Methods

### 4.1. Reagents

Lipopolysaccharide (cat.no. L2630) were from (Sigma-Aldrich, St. Louis, MO, USA), GW6471 (cat.no. 11697), GW9662 (cat.no. 70785), GSK0660 (cat.no. 15272), GW501516 (cat.no. 10004272) and fenofibrate (cat.no. 10005368) were from (Cayman Chemical, Ann Arbor, MI, USA), rosiglitazone (cat.no. R2408) were from (Sigma-Aldrich, St. Louis, MO, USA), streptomycin–penicillin (cat.no. A063), trypsin (cat.no. P037), EDTA, fetal bovine serum (cat.no BS-110/500) were from PanEco (Moscow, Russia). Culture medium Dulbecco’s Modified Eagle Medium (DMEM) (cat.no. 21885-025) (Gibco, Thermo Fisher Scientific, Waltham, MA, USA). Antibodies against COX-2 (cat.no. 12282), p38 MAPK (cat.no 9212), phospho-p38 MAPK (Thr180/Tyr182, cat.no. 9211), SAPK/JNK (cat.no. 9252), phospho-SAPK/JNK (Thr183/Tyr185, cat.no. 4668), p44/42 MAPK (Erk1/2, cat.no. 9102), phospho-p44/42 MAPK (Erk1/2, Thr202/Tyr204, cat.no. 9106) and β-actin (cat.no. 8H10D10) were from (Cell Signaling Technology, Danvers, MA, USA), secondary horseradish peroxidase conjugated antibodies (anti-rabbit, anti-mouse, and anti-goat) (SCBT and CST), western blotting substrate ECL (Thermo Fisher Scientific, cat.no 32209, Waltham, MA, USA) were used. ELISA kits for TNFα (cat.no. 558535) and IL-10 (cat.no. 555134) from BD Biosciences, San Diego, USA, San Diego, CA, USA were also used. The oxylipins standards were as follows: 6-keto PGF1α-d4 (cat.no. 315210), TXB2-d4 (cat.no. 319030), PGF2α-d4 (cat.no. 316010), PGE2-d4 (cat.no. 314010), PGD2-d4 (cat.no. 312010), 5(S)-HETE-d8 (cat.no. 334230), 12(S)-HETE-d8 (cat.no. 334570), 15(S)-HETE-d8 (cat.no. 334720) (Cayman Chemical, Ann Arbor, MI, USA). Oasis^®^ PRIME HLB cartridges (60 mg, 3cc, cat.no. 186008056) were obtained from Waters, Eschborn, Germany.

### 4.2. Primary Cell Culture

Astrocytes cell cultures were prepared as previously described [50] from newborn pups of Wistar rats. All of the experimental procedures were performed according to the guidelines in the European Convention for the Protection of Vertebrate Animals used for Experimental and Other Scientific Purposes. Effect of treatments on cell viability MTT assay was used to determine changes in viability following exposure of confluent primary astrocyte cultures to PPARs ligands and other pre- and co-treatments. No loss of viability occurred in astrocyte cultures (data not shown).

### 4.3. Western Blot Analysis

Western blot analysis was performed as described earlier [50]. In brief, cells were lysed in a modified radio immunoprecipitation assay (RIPA) buffer containing 50 mM Tris, pH 7.4, 1% NP-40 Sigma Chemicals, 0.25% Na-deoxycholate, 150 mM NaCl, 1 mM EDTA, 1 mM Na3VO4, 1 mM NaF and protease/phosphatase inhibitor cocktail (Roche Molecular Biochemicals, Mannheim, Germany). The protein concentration was determined by the standard Bradford assay. The samples were run on 10% polyacrilamide gels and the proteins were transferred to PVDF membrane. After blocking the membranes were subsequently subjected to a Phosphate-Buffered Saline with Tween 20 0.05%, with a respective primary antibody (1:1000) at 4 °C overnight with constant rotation. Secondary species-specific antibodies were applied at the concentration of 1:10,000 for 1 h at room temperature. The signals were detected using the Pierce ECL Plus Western Blotting Substrate (Thermo Scientific). Mild stripping buffer (Abcam) was used to re-probe western blot membranes. Densitometry was carried out on three different experiments. The band intensity was measured using a ChemiDoc™ XRS+ gel documentation system (Bio-Rad, Hercules, CA, USA). Densitometry was performed with ImageJ (1.51 s) software (National Institute of Health, Bethesda, MD, USA) software using β-actin as the loading control.

### 4.4. UPLC-MS/MS Conditions and Sample Preparation

After the cell experiments, the supernatant was collected and stored at −80 °C for further analysis. The cell-free culture media were taken for the solid-phase lipid extraction (Oasis^®^PRIME HLB cartridge (60 mg, 3 cc)) and analyzed using 8040 series UPLC-MS/MS mass spectrometer (Shimadzu, Japan) in multiple-reaction monitoring mode as described previously [50]. The selected lipids were identified and quantified using Lipid Mediator Version 2 software (Shimadzu, Japan).

### 4.5. Determination of TNFα, IL-10, IL-6 and Hyaluronic Acid by Enzyme-Linked Immunoassay

ELISA for cytokines was performed using the BD OptEIA, (TNFα, cat No.:558535; IL-10, cat No.:555134; BD Biosciences) antibody kits as previously described [50], following the manufacturer’s instructions. Optical density was measured using a microplate reader Synergy H4 (BioTek, Winooski, VT, USA).

### 4.6. Experimental Data Analysis and Statistics

The data are expressed as mean ± SEM. The normality of data sets was assessed using the Shapiro-Wilk test. The data were subjected to a one-way ANOVA, followed by Bonferroni’s post hoc test, in order to determine the statistical significance between groups was indicated on figures. * stands for stat sign difference between indicated bar and control cells, # stands for stat sign difference between indicated bar and LPS treated cells, ^ stands for stat sign difference between two indicated bars. The comparison was made within the corresponding kinases (Figure 5) and PPAR (Figure 4 and Figure 6). *p* < 0.05 was considered statistically significant. All of the experiments were repeated at least three times. All statistical analysis was performed using R Statistical Software (version 3.0.2.; R Foundation for Statistical Computing, Vienna, Austria).

## 5. Conclusions

Although individual oxylipins (for example, PGE2) have been studied for several decades, studies of oxylipin profiles began in the last few years with the development of mass spectrometric methods. New data change the traditional understanding of oxylipins regulation and reveal new regulation nodes in signaling cascades. This opens a new research field for the future.

The present comparison of PPARα, β, g ligands as modulators of LPS-stimulated astrocytes responses, estimated by cytokines and oxylipins release, reveal that the PPARβ ligands, primarily, agonist GW501516, are a potential pro-resolution and anti-inflammatory drug for targeting glia-mediated neuroinflammation. Moreover, GW501516 is the stronger modulator towards resolution in astrocytes (cytokine index, inhibition of prostaglandins, stimulation of HDoHEs, pro-resolution mediators, and derivatives of w-3 fatty acid). This makes PPARβ ligands potentially important therapeutic agents, especially for nervous system disorders accompanied by chronic inflammation.

## Supplementary Materials

The following are available online at https://www.mdpi.com/1422-0067/21/24/9577/s1.
