# Supplementary material for: Comparison of PPAR Ligands as Modulators of Resolution of Inflammation, via Their Influence on Cytokines and Oxylipins Release in Astrocytes
Source: Int J Mol Sci. 2020 Dec 16;21(24):9577. doi: 10.3390/ijms21249577 (PMC7765666; doi:10.3390/ijms21249577)
Supplement: Supplementary file 1 [file ijms-21-09577-s001.pdf]

SUPPLEMENTARY

Comparison of PPAR ligands as modulators of resolution of inflammation via influence on cytokines and oxylipins release in astrocytes

Dmitry V. Chistyakov [1,3,*], Alina A. Astakhova [1], Sergei V. Goriainov [3], and Marina G. Sergeeva [1]

[1] Belozersky Institute of Physico-Chemical Biology, Moscow State University, Moscow 119992, Russia; alina.an.astakhova@gmail.com (A.A.A.); mg.sergeeva@gmail.com (M.G.S.)
[2] Faculty of Bioengineering and Bioinformatics, Moscow Lomonosov State University, Moscow 119234 Russia; ridernadya@gmail.com
[3] SREC PFUR Peoples' Friendship University of Russia (RUDN University), Moscow, Russia; goryainovs@list.ru (S.V.G.), chistvic@gmail.com (V.V.C.)
* Correspondence: chistyakof@gmail.com; Tel.: +7-495-939-4332

***Corresponding authors:**

Dmitry V. Chistyakov Belozersky Institute of Physico-Chemical Biology, Lomonosov Moscow State University,1-40 Leninskye Gory, Moscow, 119992 Russia;

.

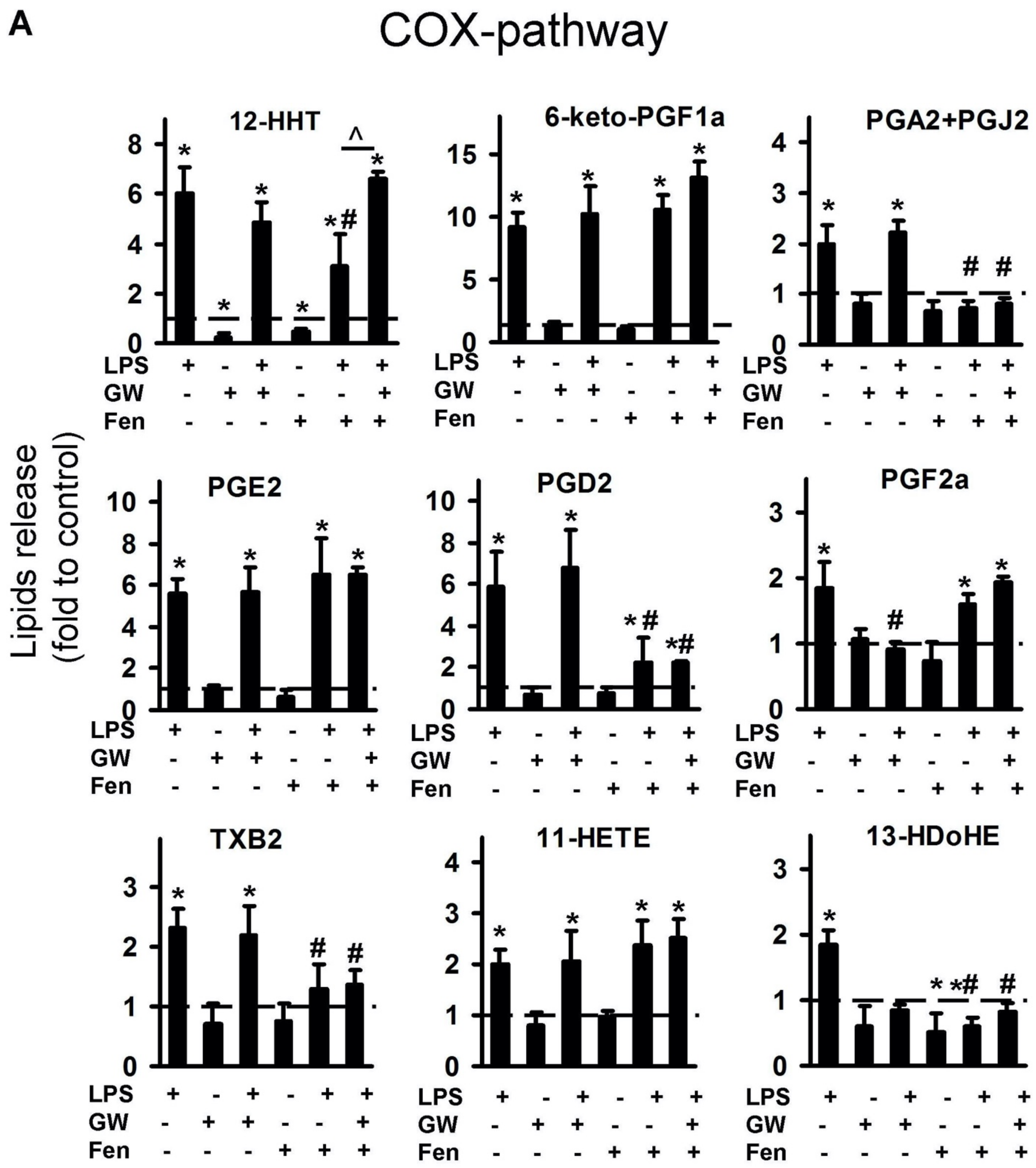
A
COX-pathway
Lipids release (fold to control)
12-HHT
6-keto-PGF1a
PGA2+PGJ2
PGE2
PGD2
PGF2a
TXB2
11-HETE
13-HDoHE
LPS
GW
Fen

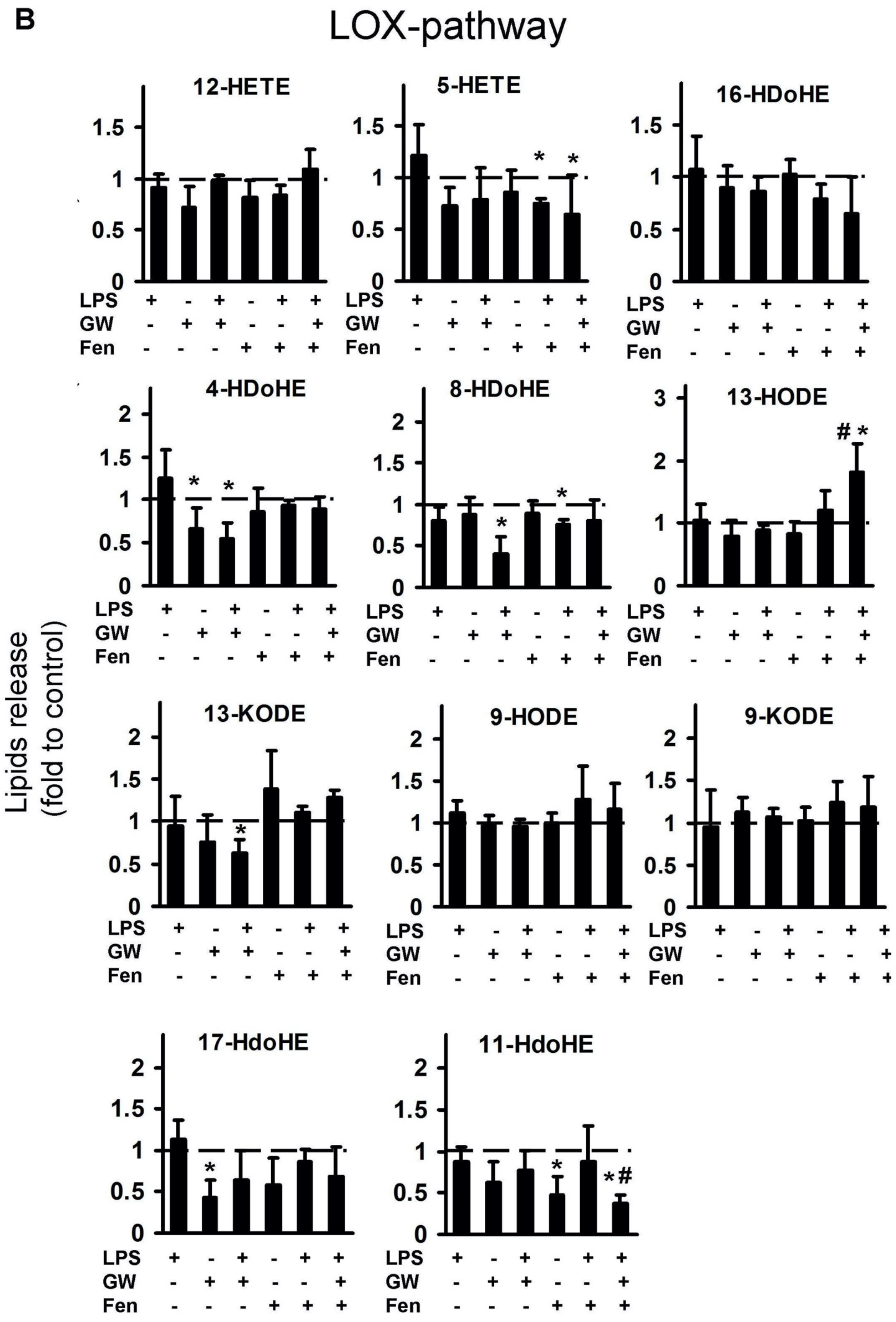

B
LOX-pathway
Lipids release (fold to control)
12-HETE
5-HETE
16-HDoHE
4-HDoHE
8-HDoHE
13-HODE
13-KODE
9-HODE
9-KODE
17-HdoHE
11-HdoHE
LPS + - + - + +
GW - + + - - +
Fen - - - + + +

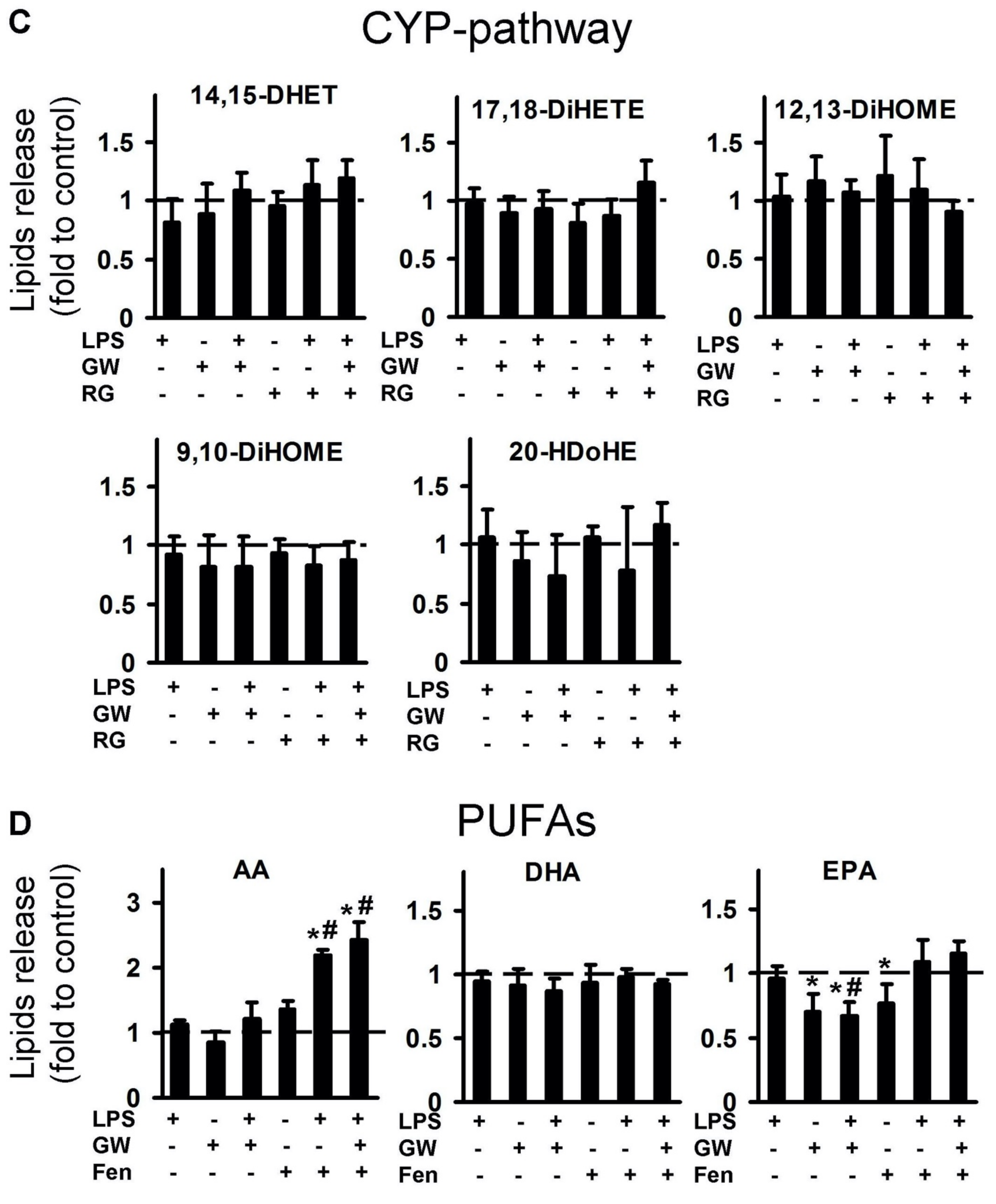

Figure S1. Effect of PPARa agonist Fenofibrate and antagonist GW6471 on the oxylipins release in the LPS-stimulated astrocytes. Primary rat astrocytes were pretreated for 30 min with GW6471 (GW6, 5 µM) or Fenofibrate (Fen, 50 µM) or in combination, and then stimulated with LPS (100 ng/mL) for 4 h. Concentrations of oxylipins in supernatants were measured using UPLC-MS/MS. The bars show relative amounts of COX-derived lipid mediators. Values represent the mean ± SEM from three independent experiments. *p<0.05, compared with the unstimulated cells, #p< 0.05, compared with the LPS-stimulated cells, ^p<0.05, compared with the indicated bars.

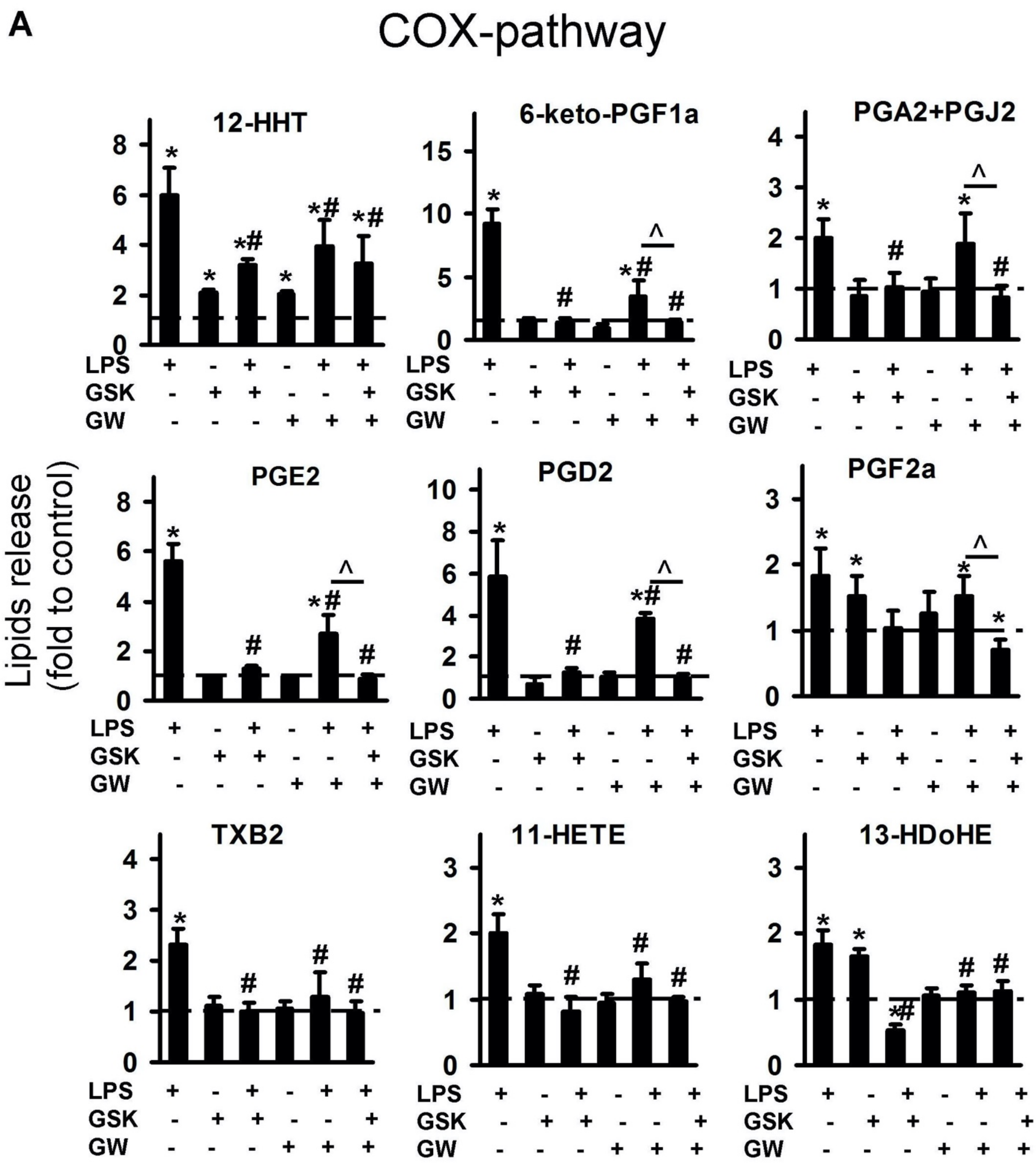
A
COX-pathway
Lipids release (fold to control)
12-HHT
6-keto-PGF1a
PGA2+PGJ2
PGE2
PGD2
PGF2a
TXB2
11-HETE
13-HDoHE
LPS
GSK
GW

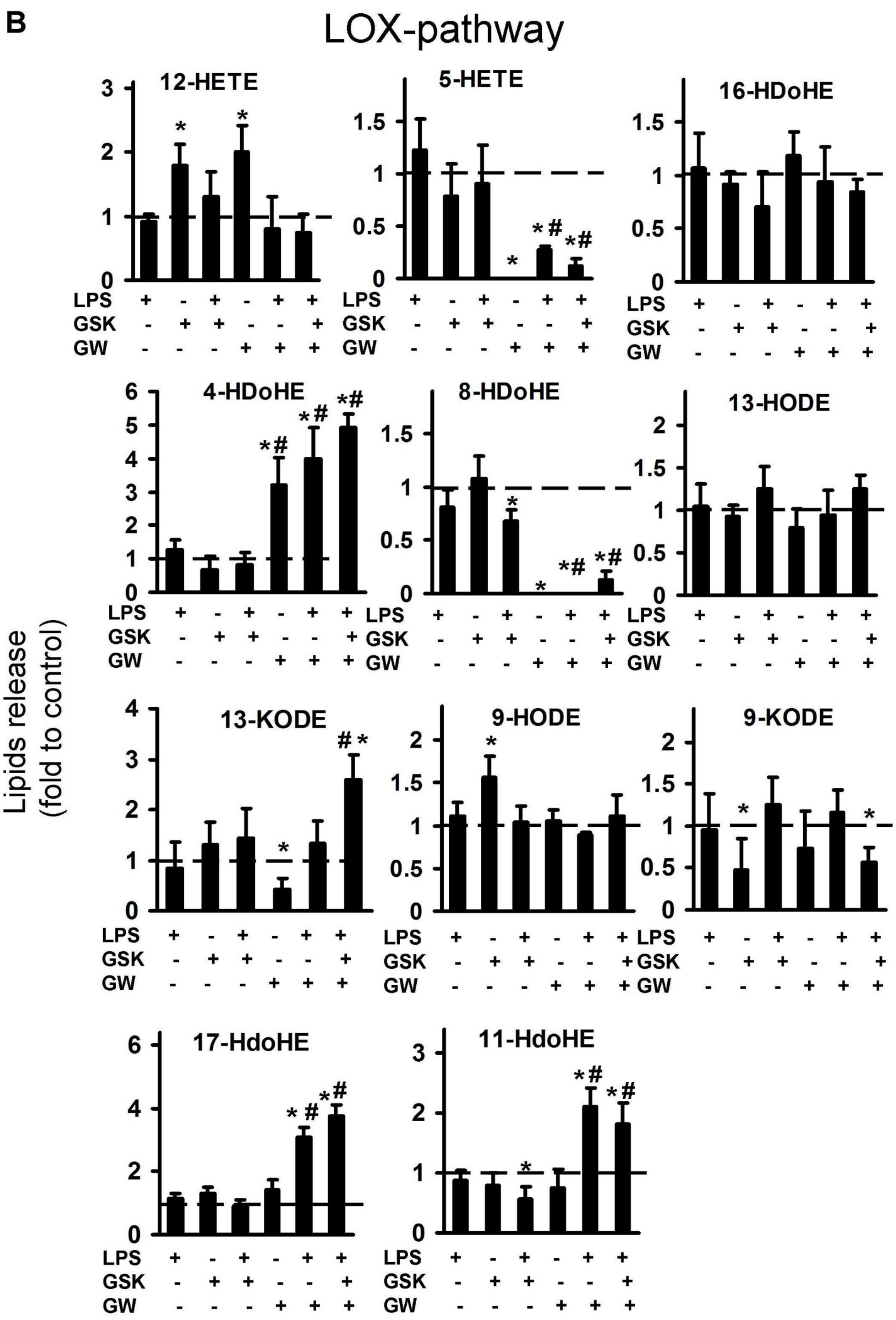

B
LOX-pathway
12-HETE
5-HETE
16-HDoHE
4-HDoHE
8-HDoHE
13-HODE
13-KODE
9-HODE
9-KODE
17-HdoHE
11-HdoHE
Lipids release (fold to control)
LPS
GSK
GW

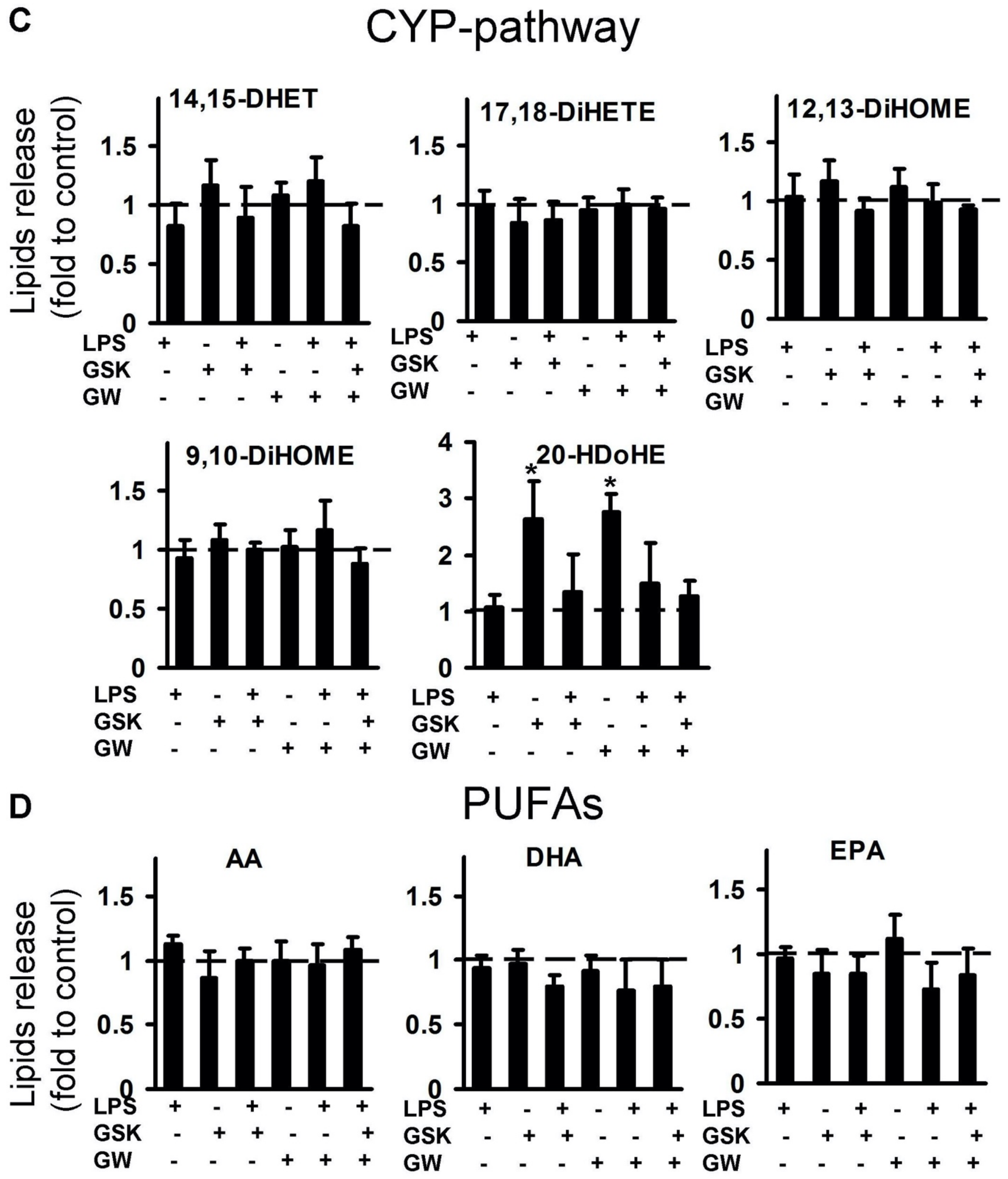

Figure S2. Effect of PPARb agonist GW501516 and antagonist GSK0660 on the oxylipins release in the LPS-stimulated astrocytes. Primary rat astrocytes were pretreated for 30 min with GSK0660 (GSK, 5 μM) or GW501516 (GW5, 25 μM) or in combination, and then stimulated with LPS (100 ng/mL) for 4 h. Concentrations of oxylipins in supernatants were measured using UPLC-MS/MS. The bars show relative amounts of COX-derived lipid mediators. Values represent the mean ± SEM from three independent experiments. *p<0.05, compared with the unstimulated cells, #p< 0.05, compared with the LPS-stimulated cells, ^p<0.05, compared with the indicated bars.

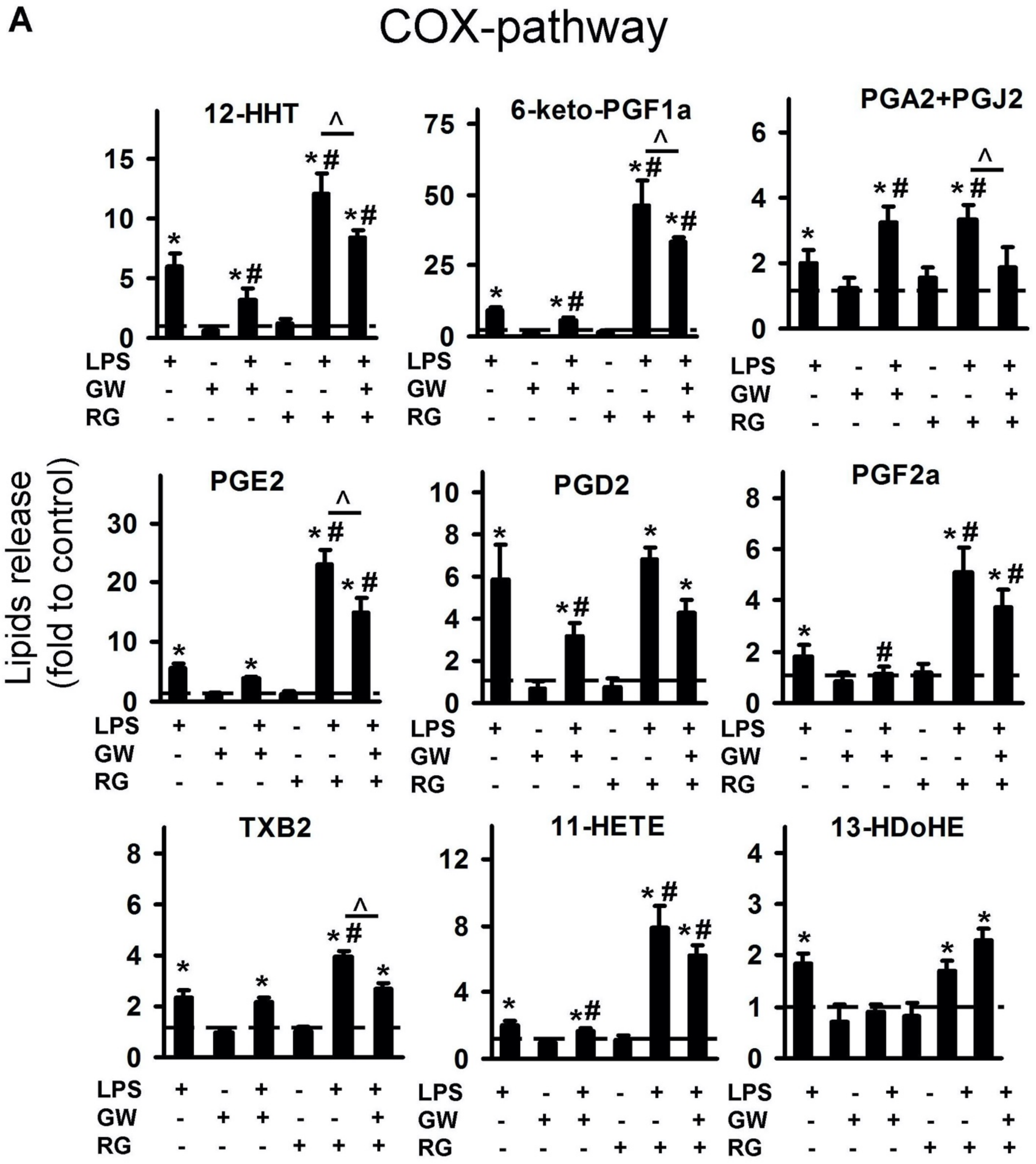
A
COX-pathway
Lipids release (fold to control)
12-HHT
6-keto-PGF1a
PGA2+PGJ2
PGE2
PGD2
PGF2a
TXB2
11-HETE
13-HDoHE
LPS
GW
RG

B

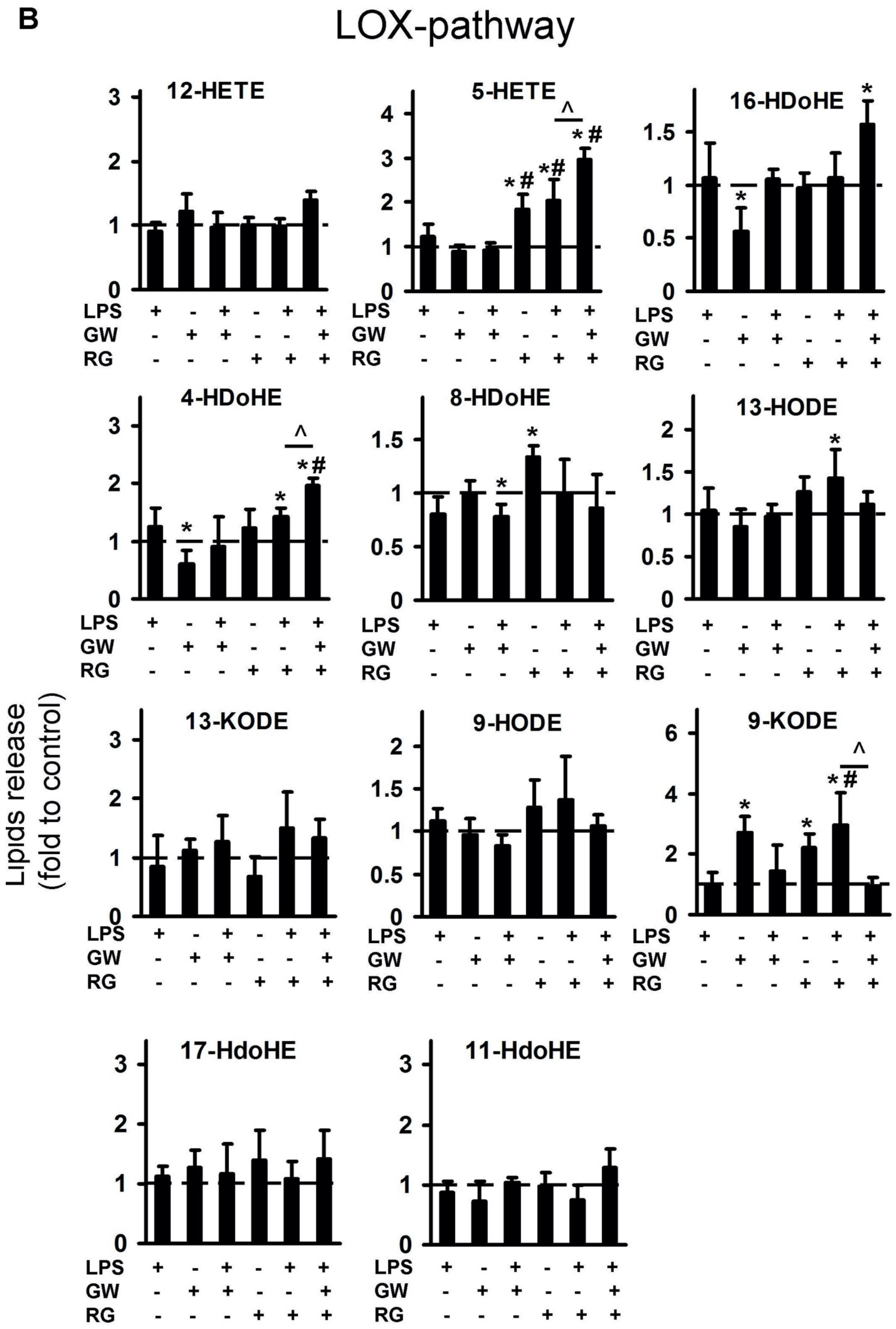
LOX-pathway
12-HETE
5-HETE
16-HDoHE
4-HDoHE
8-HDoHE
13-HODE
13-KODE
9-HODE
9-KODE
17-HdoHE
11-HdoHE
Lipids release (fold to control)
LPS + - + - + +
GW - + + - - +
RG - - - + + +

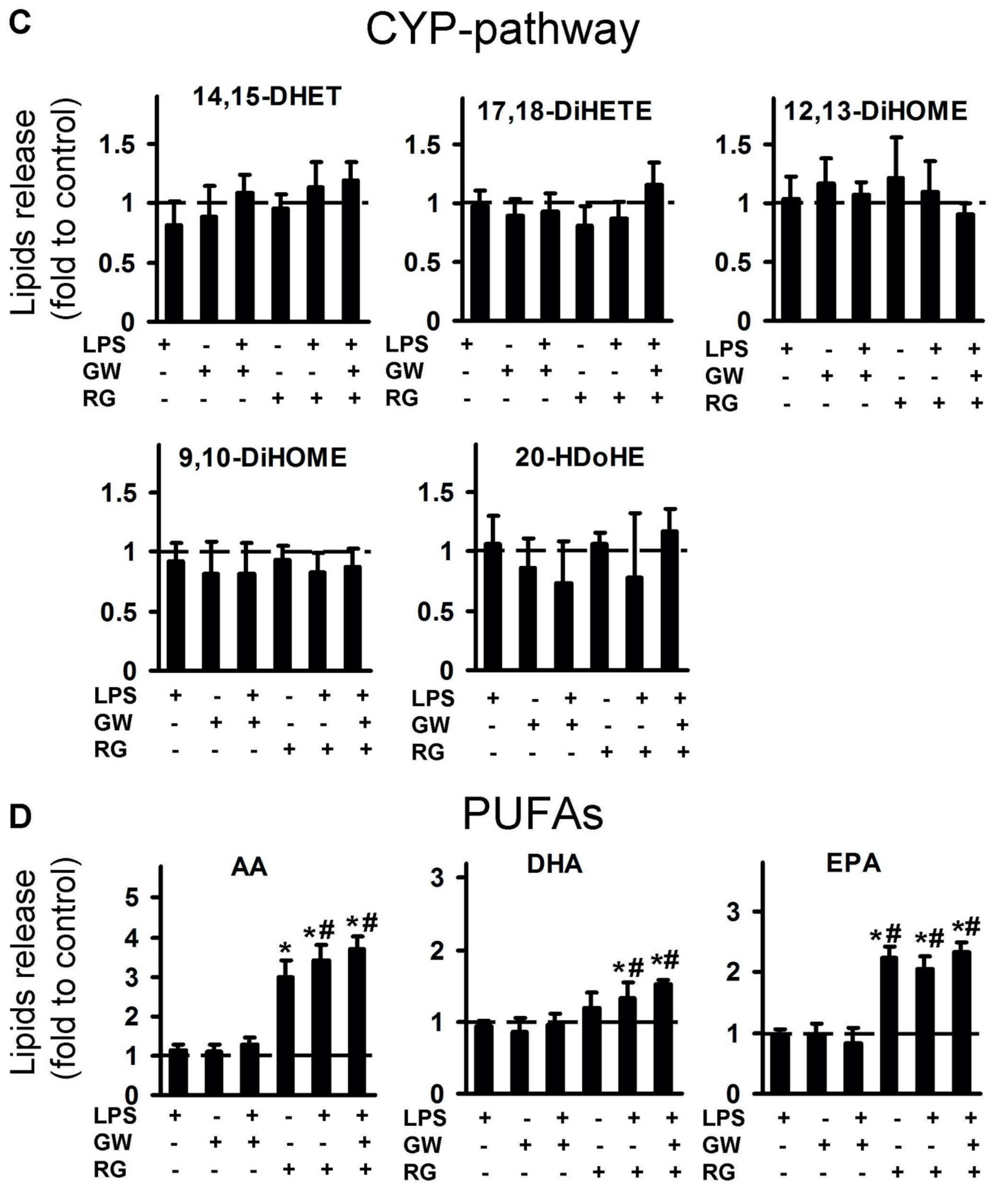

Figure S3. Effect of PPARg agonist rosiglitazone and antagonist GW9662 on the oxylipins release in the LPS-stimulated astrocytes. Primary rat astrocytes were pretreated for 30 min with GW9662 (GW9, 5 μM) or rosiglitazone (RG, 20 μM) or in combination, and then stimulated with LPS (100 ng/mL) for 4 h. Concentrations of oxylipins in supernatants were measured using UPLC-MS/MS. The bars show relative amounts of COX-derived lipid mediators. Values represent the mean ± SEM from three independent experiments. *p<0.05, compared with the unstimulated cells, #p< 0.05, compared with the LPS-stimulated cells, ^p<0.05, compared with the indicated treatment.
